# A Rare Case of Massive Ovarian Mucinous Cystadenoma With Postmenopausal Bleeding

**DOI:** 10.7759/cureus.10198

**Published:** 2020-09-02

**Authors:** Manoj R Somagutta, Enkhmaa Luvsannyam, Molly S Jain, Krystel Elliott-Theberge, Amit S Grewal, Siva K Pendyala, Charles Edwards

**Affiliations:** 1 Medicine, California Institute of Behavioral Neurosciences and Psychology, Fairfield, USA; 2 Internal Medicine, Saint James School of Medicine, Park Ridge, USA; 3 Obstetrics and Gynecology, Saint James School of Medicine, Park Ridge, USA; 4 Obstetrics and Gynecology, Amita Health Adventist Medical Health Center Hinsdale, Hinsdale, USA; 5 Obstetrics and Gynecology, Avalon University School of Medicine, Willemstad, CUW

**Keywords:** ovarian mass, cystic mass, mucinous cystadenoma, abdominal distension, abdominal pain

## Abstract

Mucinous cystadenomas are among the most common benign ovarian neoplasms. They are known for their massive size causing compressive effects ranging from pressure, pain, bloating, and urinary symptoms. Over time, these adnexal masses can lead to fatal complications, such as ovarian torsion or hemorrhage. Incidental findings of these tumors are common as many of these patients are asymptomatic. Pelvic examinations and imaging studies can be used to further diagnose symptomatic patients and aid physicians in developing an appropriate course of treatment. We report a rare case of a large mucinous cystadenoma, with a size of 25 × 25 cm and concurrent management of postmenopausal bleeding. We present the data from the admission of the patient to her discharge, including history and physical examination, diagnostic reports, transabdominal ultrasound, CT scan, surgical evaluation, and surgical-pathology reports. Abdominal pain can present in a variety of different scenarios, and ovarian masses only represent a small portion of the differentials. Mucinous cystadenomas constitute an even smaller percentage of these ovarian growths. As discussed in this case report, a large ovarian mucinous cystadenoma was compressing the surrounding structures resulting in a wide array of symptoms. The case describes the importance of extensive diagnostic evaluation and prompt surgical management of these ovarian tumors. It also brings attention to the significance of diagnosing a medical condition such as postmenopausal bleeding promptly to avoid potential negative outcomes.

## Introduction

Ovarian cysts occur commonly in women of childbearing ages [1]. They are usually benign and therefore tend to be asymptomatic in the majority of the population. The differential diagnoses of benign ovarian cysts include dermoid cysts, Brenner cysts, and mucinous cysts. Dermoid cysts are sac-like growths on ovaries containing fat, hair, and other tissue types. Brenner cysts are found incidentally and are solid outgrowths on the ovarian surface. Of all the ovarian tumors, mucinous cystadenomas account for 15%-20% [2]. These tumors arise from the ovarian surface epithelium and have smooth inner and outer thin walls. Specifically, benign mucinous cystadenomas comprise 80% forming the majority of ovarian mucinous tumors; 10% of these tumors are found to be malignant and the remaining 10% are borderline [3]. A striking feature for these benign mucinous cystadenomas is that they could become massive in size ranging from 5 to 28 cm with larger size increasing the risk of malignancy [4]. When cysts begin to enlarge and invade surrounding structures, a patient may begin to present with varying symptoms of a mass effect from mild pain or pressure to severe life-threatening complications, such as ovarian torsion, cystic rupture, and hemorrhage [5].

## Case presentation

**Figure 1:**
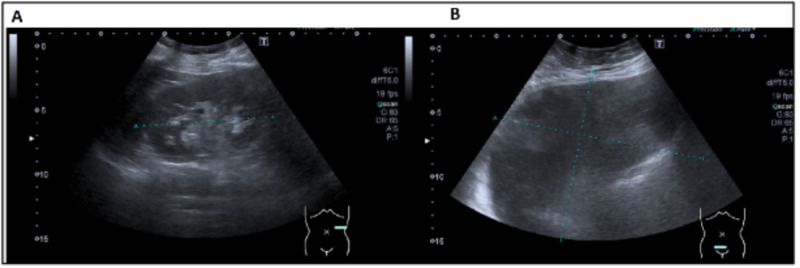
Transabdominal ultrasonography showing a transverse view of the left upper quadrant (A) and lower abdomen (B) of a cystic lesion.

This report involves a 59-year-old female who presented with postmenopausal uterine bleeding, increased abdominal girth, and discomfort. The patient was a para 1, postmenopausal Afro-Curaçaoan woman who was referred to our department after presenting with postmenopausal bleeding (PMB), abdominal distention for one month, and worsened abdominal pain for two months. The patient’s symptoms were associated with lower back pain, swelling of her feet, constipation with excessive straining, dysuria, urinary frequency, and urgency. The patient also complained of painful intercourse and postcoital bleeding. She denied nausea, vomiting, dizziness, or diarrhea. She denied a history of liver disease or similar episodes in the past. Her past medical history is significant for a single cerebrovascular accident nine years ago and epilepsy. The patient had one cesarean section at the age of 33 years, cataract surgery 11 years prior, and cholecystectomy 6 years before the current presentation. She reported that her menarche was at the age of 11 years, with regular menstrual cycles lasting for five days. The patient had a pap smear one year before the current visit, and the results were normal. The patient did not take any regular medications, including hormonal therapy. She had no family history of the bowel, breast, ovarian, and uterine cancer. Physical examination revealed abdominal distension with a large mass of 35 cm above the pubic symphysis. There was no fluid wave thrill, no focal tenderness, rebound, or guarding of the abdomen upon examination. Normal cervix with vaginal atrophy was noted. Her blood work was unremarkable. A transabdominal ultrasound scan (USS) showed a cyst with no solid components with minimal free intraperitoneal fluid (Figure 1).

**Figure 2:**
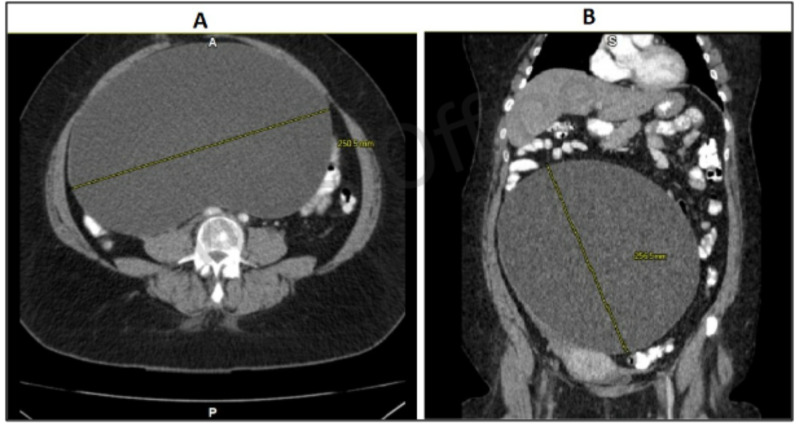
Abdominal CT in axial (A) and coronal (B) sections showing large, well-defined, unilocular cystic mass measuring 25 × 25 cm.

Abdominal CT scan confirmed a large cystic mass, measuring 25 × 25 cm, causing compression around the surrounding organs (Figure 2). 

Endometrial dilation and curettage revealed a moderate amount of tissue that was sent for pathological evaluation, and the result showed polypoid endometrium with no hyperplasia and no malignancy.

**Figure 3:**
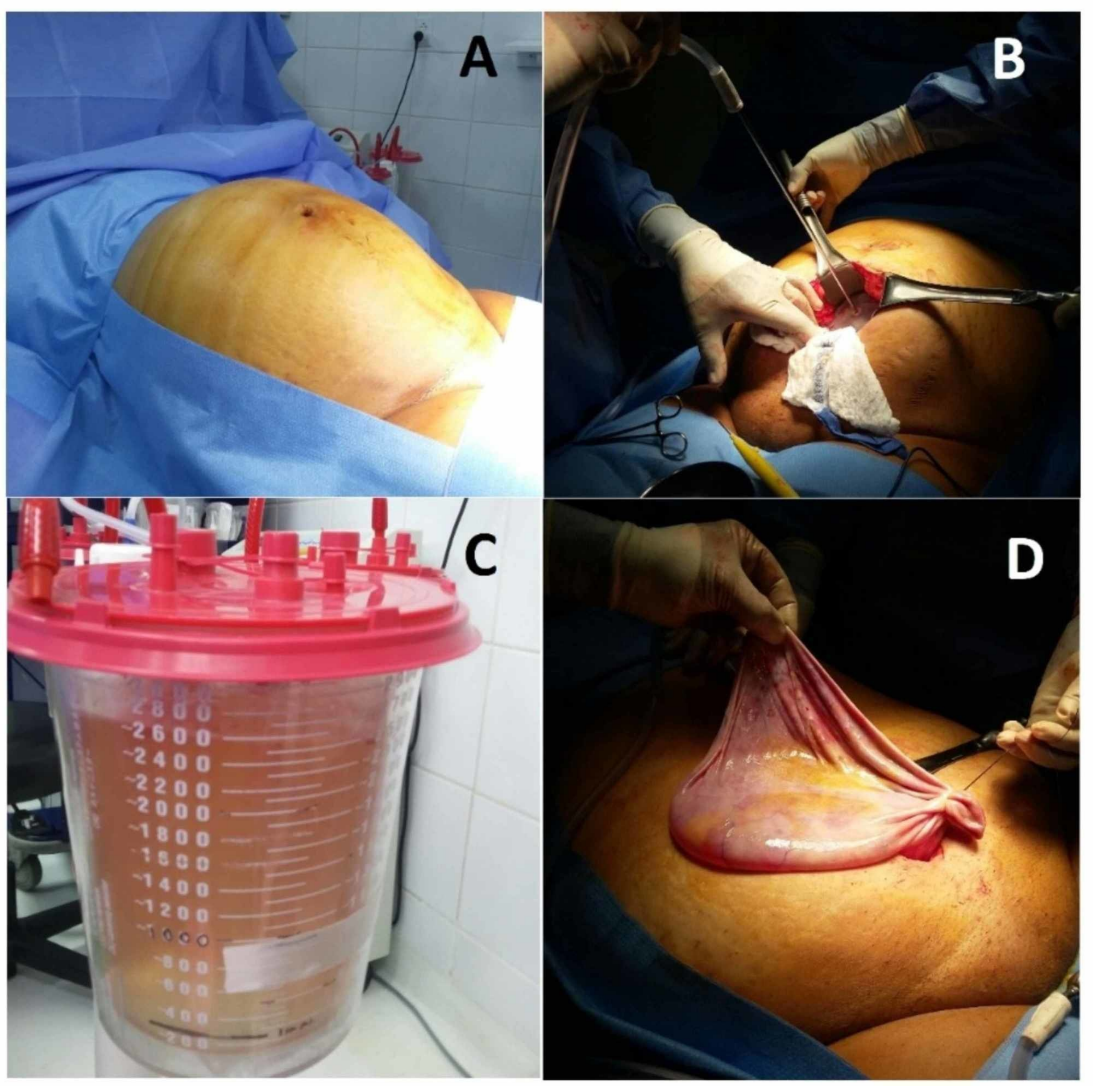
Abdominal distension of the patient before surgery (A), cyst fluid is drained with a large needle and suction tube (B, C), right ovarian cyst after fluid drainage measuring 18 × 16 cm (D).

One month later, the exploration of the abdomen through a small vertical incision revealed a massive right ovarian cyst, smooth and gray in color, occupying the entire pelvic and abdominal cavities up to the xiphoid process. No pelvic or para-aortic nodularity was palpated, and no ascites were noted. Approximately 2,600 ml of clear fluid was aspirated from the ovarian cyst followed by a total abdominal hysterectomy with bilateral salpingo-oophorectomy and right ovarian cystectomy (Figure 3). The tissue was sent for pathological evaluation and was subsequently found to have mucinous cystadenoma and uterine leiomyoma with adenomyosis. Surgical drainage with the removal of the cyst from the abdominal cavity and total hysterectomy with bilateral salpingo-oophorectomy resulted in complete resolution of the patient's symptoms.

## Discussion

There are different subtypes and tumor cell origins that lead to ovarian neoplasms. These include surface epithelium, germ cell, or sex cord-stromal tissue [6]. The majority of the diagnosed ovarian neoplasms are benign, and 95% of all gynecological malignancies are due to epithelial cells. Of all the ovarian epithelial cell cysts, 80% of ovarian mucinous cystadenomas are benign and 5%-10% present in bilateral ovaries [7]. 

The size and laterality play a major role in the determination of tumor origin whether primary or metastatic. In contrast with metastatic tumors, the primary tumors tend to be larger and unilateral [4]. The large size of the ovarian cyst is highly suggestive of mucinous histology due to mucus filling the cyst. Likewise, in this case, the patient’s abdominal CT scan confirmed a large cystic mass measuring 25 × 25 cm. This massive size can fill up the entire abdominal and pelvic cavity, resulting in compressive symptoms ranging from ureteral obstruction to abdominal compartment syndrome. Our patient presented with severe abdominal distension with varying symptoms, including urinary symptoms, lower back pain, lower extremity edema, dyschezia, and dyspareunia. 

The initial diagnosis of adnexal mass is obtained by the pelvic ultrasound (US). In particular, the transabdominal US and the transvaginal US become essential tools for evaluating ovarian masses [1]. CT scan and MRI can further help in visualizing the specificity of ovarian cysts. Cancer antigen 125 (CA-125) is an important tumor marker that helps differentiate between benign and malignant ovarian masses [1]. Hence, it is ideal to offer early screening to high-risk women with a family history of ovarian cancer using the tumor marker CA-125 and the transvaginal US that can aid in early diagnosis of this benign adenoma [6]. Kirsten rat sarcoma viral oncogene homolog (KRAS) mutations occur inside the RAS family of G proteins, which signal cell division; such changes promote cell development and are altogether expanded in mucinous ovarian tumors, including mucinous cystadenomas and adenocarcinomas [4]. 

Management of ovarian masses depends on a combination of factors, such as age, medical history, symptoms, size of the cyst, and menopausal state of the patient. The mucinous cyst rupture can lead to mucinous deposits filling in the entire peritoneum as the complication commonly called *pseudomyxoma peritonei* [7]. The best treatment is unilateral salpingo-oophorectomy or ovarian cystectomy with the removal of the adnexal mass [1]. However, performing surgical interventions for large masses, as in our patient, is often associated with fatal consequences, such as sepsis, pulmonary embolism, and cardiac failure [2]. Therefore, appropriate monitoring and follow-up are highly recommended in the postoperative period. 

Our patient also presented with PMB with the pathological findings of intramural leiomyoma of the uterus with adenomyosis. Adenomyosis occurs when the healthy uterine tissue starts growing inside the uterine muscular wall, and intramural leiomyoma of the uterus, also known as fibroid, is a benign tumor growing in between the uterine muscles [8]. Having unresolved adenomyosis after menopause is unique to this case because it usually spontaneously resolves during the postmenopausal period [9]. There is a likelihood that our patient had undiagnosed adenomyosis before menopause or they discovered accidental samples for adenomyosis in pelvic tissue after the medical procedure. Uterine fibroids are the most common causes of heavy uterine bleeding and may cause pelvic issues when complicated. Furthermore, our patient’s pathological report suggested polypoid endometrium without any signs of hyperplasia or malignancy. Uterine polyps are hyperplastic overgrowths of endometrial glands and stroma projecting from the inner wall lining of the uterine cavity. They are usually benign and mostly occur in women completing menopause [8]. They are also commonly involved in causing abnormal uterine bleeding, another possible cause for the PMB seen in our patient.

## Conclusions

An adnexal mass is a common gynecological problem found in women of all ages, from fetuses to older adults. Mucinous cystadenoma, a benign neoplasm, has the potential to grow more extensively than other ovarian tumors causing symptoms owing to mass effect as discussed in our patient. It was managed through surgical resection and drainage of fluid. Hence, it is prudent for physicians to perform imaging and routine check-ups targeted at early detection to prevent morbidity associated with these massive tumors. The importance of patients reporting mild to moderate symptoms can prevent future fatal complications as seen in our patient with PMB due to undiagnosed adenomyosis. This case also highlights significant causes of PMB other than endometrial cancer that clinicians need to rule out in their diagnostic process. 
